# A Machine Learning Approach to Investigate the Uncertainty of Tissue-Level Injury Metrics for Cerebral Contusion

**DOI:** 10.3389/fbioe.2021.714128

**Published:** 2021-10-08

**Authors:** Andrea Menichetti, Laura Bartsoen, Bart Depreitere, Jos Vander Sloten, Nele Famaey

**Affiliations:** ^1^ Biomechanics Section, Department of Mechanical Engineering, KU Leuven, Leuven, Belgium; ^2^ Neurosurgery, University Hospitals Leuven, Leuven, Belgium

**Keywords:** brain biomechanics, cerebral contusion, finite element modeling, tissue-level injury criteria, machine learning, controlled cortical impact, traumatic brain injury

## Abstract

Controlled cortical impact (CCI) on porcine brain is often utilized to investigate the pathophysiology and functional outcome of focal traumatic brain injury (TBI), such as cerebral contusion (CC). Using a finite element (FE) model of the porcine brain, the localized brain strain and strain rate resulting from CCI can be computed and compared to the experimentally assessed cortical lesion. This way, tissue-level injury metrics and corresponding thresholds specific for CC can be established. However, the variability and uncertainty associated with the CCI experimental parameters contribute to the uncertainty of the provoked cortical lesion and, in turn, of the predicted injury metrics. Uncertainty quantification *via* probabilistic methods (Monte Carlo simulation, MCS) requires a large number of FE simulations, which results in a time-consuming process. Following the recent success of machine learning (ML) in TBI biomechanical modeling, we developed an artificial neural network as surrogate of the FE porcine brain model to predict the brain strain and the strain rate in a computationally efficient way. We assessed the effect of several experimental and modeling parameters on four FE-derived CC injury metrics (maximum principal strain, maximum principal strain rate, product of maximum principal strain and strain rate, and maximum shear strain). Next, we compared the *in silico* brain mechanical response with cortical damage data from *in vivo* CCI experiments on pig brains to evaluate the predictive performance of the CC injury metrics. Our ML surrogate was capable of rapidly predicting the outcome of the FE porcine brain undergoing CCI. The now computationally efficient MCS showed that depth and velocity of indentation were the most influential parameters for the strain and the strain rate-based injury metrics, respectively. The sensitivity analysis and comparison with the cortical damage experimental data indicate a better performance of maximum principal strain and maximum shear strain as tissue-level injury metrics for CC. These results provide guidelines to optimize the design of CCI tests and bring new insights to the understanding of the mechanical response of brain tissue to focal traumatic brain injury. Our findings also highlight the potential of using ML for computationally efficient TBI biomechanics investigations.

## Introduction

Cerebral contusion (CC) is a common type of traumatic brain injury (TBI) found across all age groups, which is often associated with lifelong disability and mortality (Alahmadi et al., 2010; Kirkman et al., 2013; Melvin and Yoganandan, 2015). CC is a focal TBI occurring from direct impacts and consisting of a bruise on the brain’s surface (Hardman and Manoukian, 2002). CC is pathologically characterized by hemorrhagic lesions accompanied by necrosis and edema that generate within the cortex. In severe cases, CC can develop in the subcortical white matter, which may require immediate surgical intervention (McGinn and Povlishock, 2016). Besides falls and assaults, accidents on public roads are a major cause of CC (Ratnaike et al., 2011). Depreitere et al. (2004) found that 73% of the victims of bicycle-related casualties were diagnosed with CC. There is still no consensus on the mechanism causing CC, although a few theories have been proposed; these include the cavitation arising from the negative pressures generated at the opposite side with respect to the impact location (Gross, 1958) and the shear strain at bony protuberances consequent to the relative motion between skull and brain tissue (Holbourn, 1943), which could explain why the majority of CC are observed in the frontal and temporal lobes (Ommaya and Ommaya, 1995; Depreitere et al., 2004; Ratnaike et al., 2011).

Controlled cortical impact (CCI) is commonly used as a model of brain trauma to investigate the mechanopathology of CC *in vivo* on different animal species, including ferret, rodent, swine, and nonhuman primates (Xiong et al., 2013; Osier and Dixon, 2017; Kinder et al., 2019). In CCI experiments, a pneumatically or electromagnetically driven piston delivers the impact on the animal’s exposed brain with a preestablished velocity and depth of indentation, therefore inducing a reproducible and localized injury. The swine model has been largely used because of the similarities with humans in terms of pathological features (Kinder et al., 2019) and brain tissue’s mechanical properties (MacManus et al., 2020). However, a large span of values has been reported in the literature for experimental parameters, including but not limited to, the geometrical characteristics of the impactor or the depth and velocity of impact (Duhaime et al., 2000; Alessandri et al., 2003; Manley et al., 2006; Meissner et al., 2011; Sindelar et al., 2017; De Kegel et al., 2021). The variability and the uncertainty of the experimental parameters all contribute to the uncertainty in the injury outcome provoked by CCI.

Computational models of the head have been extensively used to quantify in mechanical terms the intracranial response to TBI by analyzing the relationship between the functional/structural damage and the brain tissue’s stress and strain fields (Chen et al., 2014). In particular, finite element (FE) models have been regarded as valuable tools to determine tissue-level local injury thresholds for specific types of TBI (Kleiven, 2007; Scott et al., 2016; Giordano et al., 2017; Zhao et al., 2017; Horstemeyer et al., 2019; Zhou et al., 2019; Hajiaghamemar et al., 2020; Trotta et al., 2020; Wu et al., 2021). Regarding CC, several injury metrics have been proposed based on different quantities, with lack of agreement on which criterion outperforms the others. These metrics include maximum principal strain (Shreiber et al., 1997; Miller et al., 1998; Viano and Lövsund, 1999; Mao et al., 2010b; Mao and Yang, 2011), shear strain (Mao and Yang, 2011), strain rate (Viano and Lövsund, 1999; King et al., 2003), product of strain and strain rate (Viano and Lövsund, 1999; King et al., 2003), strain energy density (Shreiber et al., 1997; Mao and Yang, 2011), von Mises stress (Shreiber et al., 1997; Miller et al., 1998), maximum principal stress (Shreiber et al., 1997), shear stress (Huang et al., 2000), and intracranial pressure (Miller et al., 1998; Huang et al., 2000; Takhounts et al., 2003; Mao and Yang, 2011; Mao et al., 2013). In the case of focal injury patterns resulting from, for example, CCI or dynamic vacuum pressure tests, the use of strain and strain rate injury criteria is preferred over other metrics assessed in the literature, such as those based on stress, intracranial pressure, or strain energy (Shreiber et al., 1997; Mao and Yang, 2011). While several FE models of the pig brain have been developed to investigate different diffuse TBI scenarios (Coats et al., 2012; Zhu et al., 2013; Yates and Untaroiu, 2016; Hajiaghamemar and Margulies, 2021; Wu et al., 2021), no computational model has specifically targeted the localized brain response to CCI experiments.

A recognized disadvantage of FE head models that have a high degree of geometrical complexity and biofidelity is the significant simulation runtime (Takhounts et al., 2008; Wu et al., 2020; Ghazi et al., 2021; Zhan et al., 2021) even when using high-performance computing platforms. This hampers the feasibility of studies that require repeated simulations, such as design optimization workflows, parameter estimation, or uncertainty quantification analyses. For the latter category, probabilistic methods are commonly used in biomechanics (Pal et al., 2008; Laz and Browne, 2010; Strickland et al., 2010; Bartsoen et al., 2021). Among them, the Monte Carlo simulation (MCS) is regarded as the gold standard technique because of its robustness and ability to converge to the correct uncertainty distribution solution (Laz and Browne, 2010). However, MCS is a time-consuming technique since it involves random sampling of the complex model, for example, the FE porcine brain model, depending on the statistical distribution of the input parameters. The accuracy of the solution (i.e., the uncertainty of the outcome of the model) depends on the number of samples considered, which can lead to computationally costly analyses. A time-efficient alternative is to use surrogates of the computationally expensive FE models. The development and implementation of supervised machine learning (ML)–based surrogates of complex biomechanics models is relatively recent. Promising applications of the ML surrogates include parameter estimation and uncertainty quantification problems across several fields, such as TBI (Cai et al., 2018; Wu et al., 2019; Ghazi et al., 2021; Schroder et al., 2021; Zhan et al., 2021), cardiovascular (Davies et al., 2019; Cai et al., 2021) and musculoskeletal biomechanics (Pal et al., 2008; Strickland et al., 2010; Bartsoen et al., 2021). Regarding the research on head impact biomechanics, Wu et al. (2019), Ghazi et al. (2021), Zhan et al. (2021) employed machine learning algorithms to predict the brain strain response of computationally expensive FE models of the human head in an accurate and time-efficient way. Once trained and tested, the ML surrogates can estimate the outcome of the complex model in a conveniently short time frame. In the case of uncertainty quantification problems, an MCS can be directly performed on the ML surrogate outcome with the advantage of reducing dramatically the computational cost, albeit accepting a degree of approximation (Laz and Browne, 2010).

This study has four main outcomes. First, we present the FE model of an atlas-based porcine brain undergoing CCI, which predicts the mechanical response of the brain tissue to the impact. Second, we develop an artificial neural network as a computationally efficient ML surrogate of the FE pig brain model. Third, by means of the ML surrogate model, we evaluate the effect of different experimental and modeling parameters on four potential CC injury metrics, that is, maximum principal strain, maximum principal strain rate, product of maximum principal strain and strain rate, and maximum shear strain. Finally, we compare the experimental cortical injury data from *in vivo* CCI tests on pig brain with the *in silico* results in order to evaluate the prediction performance of the tissue-level injury metrics specific for CC.

## Materials and Methods

### Controlled Cortical Impact Tests

**FIGURE 1:**
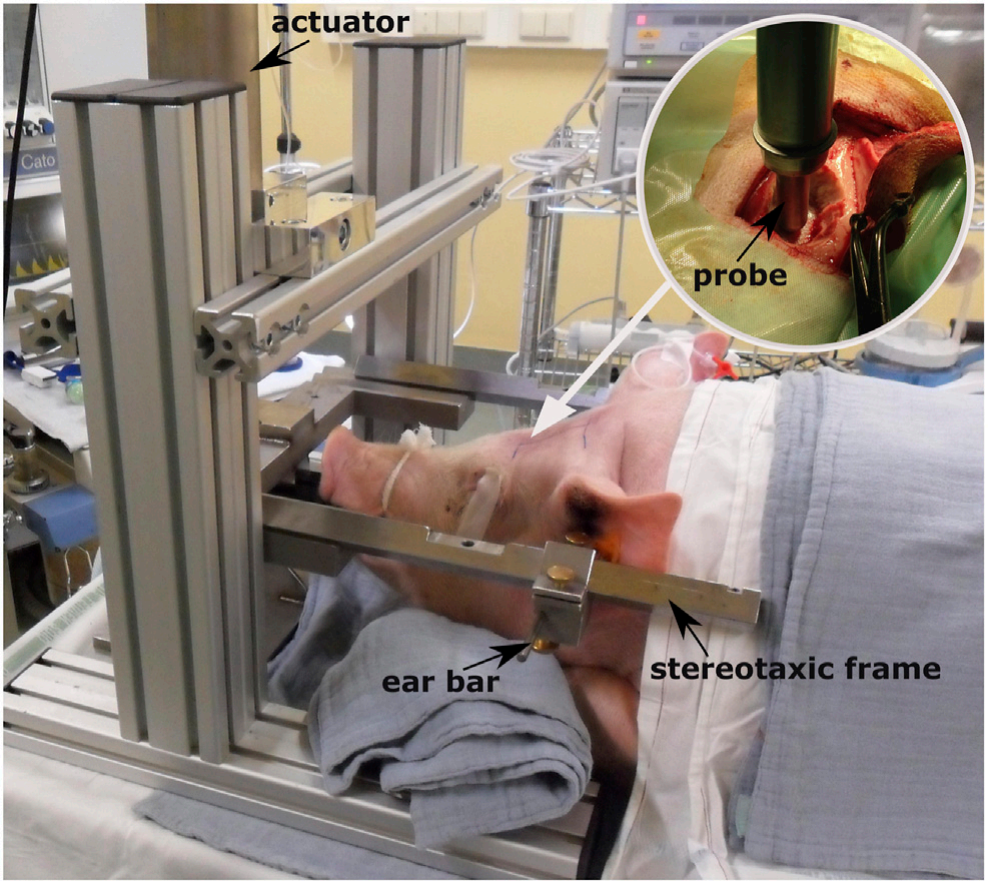
Controlled cortical impact setup [adapted from De Kegel (2018)] and positioning of the animal prior to testing. The white arrow indicates the point where the craniotomy is performed after sedating the animal. The circular image on the top right corner illustrates how the probe is mounted on the electromagnetically driven actuator and is placed over the closed dura mater before the impact.

The experimental data on the assessment of CC damage were retrieved from a previous study within our research group, where 34 Landrace male domestic pigs, aged 4–5 months, and weighing on average 60.7 ± 10.9 kg underwent controlled cortical impacts *in vivo* (Figure 1). The full description of the experimental protocol can be found in De Kegel et al. (2021) and it is briefly summarized here. General anesthesia was induced to the animals prior to performing craniotomy, which was done unilaterally on the left hemisphere and was located immediately parasagittal and posterior to the coronal suture. Afterward, the impact was delivered directly on the exposed dura mater by means of an electromagnetically controlled cortical impactor (PinPoint PCI3000, Hatteras Instrument Inc.), which regulated the impact depth (range between 1.1–12.6 mm), velocity (range between 0.4–2.2 m/s), and dwell time (= 200 ms) of an hemispherical stainless steel tip (diameter = 10 mm). A brain magnetic resonance (MR) scan (Prisma Fit 3T scanner, Siemens) was performed under general anesthesia 48 h after the impact to ensure full development of CC. The presence of cortical damage and underlying white matter edema were evaluated on the MR scans by a senior neuroradiologist and a senior neurosurgeon. The volume of white matter edema was quantified using image segmentation (Mimics, Materialise). After the scans, the animals were euthanized and the brain was extracted for histology processing with the aim of confirming the presence of cortical necrosis, an indicator of the provoked CC. Table 1 summarizes the combination of impact parameters chosen and the cortical damage evaluation in the 14 cases of the cohort for which full CC assessment was possible.

**TABLE 1:** Experimental impact parameters and results of the CCI performed *in vivo* on the pig brain (De Kegel et al., 2021) for which medical imaging and histological assessment of CC were available. In case of detectable disruption of the cortical layer the “Cortical damage” is marked as “ + ,” otherwise as “ −. ” The animal number in parentheses refers to the numbering used in De Kegel et al. (2021).

Animal #	Peak depth (mm)	Velocity (m/s)	Cortical damage
I (12)	11.2	2.1	+
II (13)	8.9	2.2	−
III (14)	9.1	1.8	+
IV (15)	10.8	1.8	+
V (16)	10.8	1.8	+
VI (17)	12.6	1.7	+
VII (18)	8.9	2.2	+
VIII (19)	6.9	2.2	+
IX (20)	5.5	2.0	+
X (21)	4.3	1.5	−
XI (22)	2.3	0.8	−
XII (31)	2.0	0.4	−
XIII (33)	1.1	0.4	−
XIV (34)	2.8	0.4	+

### Development and Validation of the FE Model of the Porcine Brain Undergoing CCI

The FE model mimics the experimental loading conditions that the porcine brains underwent during CCI testing. The model consists of the impacting probe, the skull, the meningeal complex, the cerebrospinal fluid, and the brain. The geometry of the brain was generated in 3-Matic (Materialise) after segmentation in Mimics (Materialise) using a high voxel resolution (100 μm × 150 μm × 100 μm) MR image-based 3D atlas of the domestic pig’s brain (Saikali et al., 2010). The obtained mask was smoothed while preserving the gyral pattern. The 3D atlas-based geometry was rescaled by a factor of 1.27 to match the average brain size of the pigs of the experimental cohort, calculated by measuring the anteroposterior length of the corpus callosum in the sagittal plane of every post-CCI MR scan. Since the standard deviation of the scaling factors was relatively low (= 0.07), we used a single brain size for all CCI cases. In order to reduce the computational cost, only the left hemisphere was considered for the model. This is a fair approximation, justified by the fact that unilateral CCI has a negligible effect on the unimpacted hemisphere (Elliott et al., 2008; Mao and Yang, 2011). The two outermost meningeal membranes (i.e., the dura-arachnoid mater (DAM)) were modeled as a unique part, while the third and innermost layer (the pia mater) was integrated into the brain part, in a similar fashion as MacManus et al. (2017). The DAM geometry was generated in 3-Matic by offsetting the cortical surface of the brain outwards and subsequently wrapping and smoothing the brain convolutions. Similar to Coats et al. (2012), an average gap of 1 mm was maintained between the surface of the brain and the inner surface of the DAM to account for the subarachnoid space in which the cerebrospinal fluid (CSF) flows.

**FIGURE 2:**
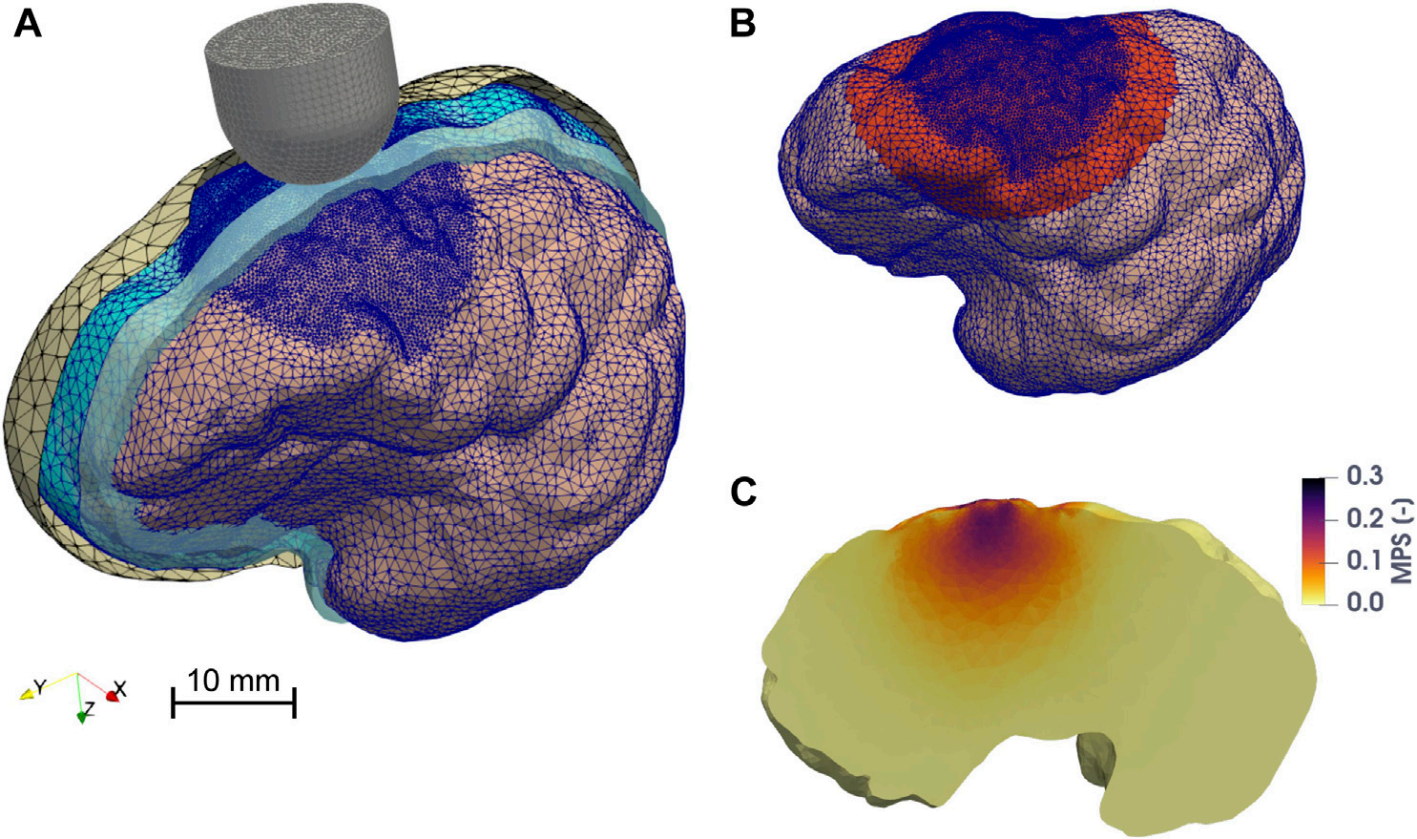
Finite element model of the porcine brain undergoing CCI. **(A)** Overview of the assembly of the model. The skull, the DAM, and the CSF are represented with parallel sectioned views. The mesh of the discrete rigid parts (skull = yellow and probe = gray) is depicted in black, while the mesh of the deformable bodies (brain hemisphere = pink, DAM = cyan), which is finer in the vicinity of the impact point, is colored in blue. The cavity filled by CSF is represented in light blue. **(B)** The zone highlighted in red represents the elements of the brain (in the undeformed configuration) belonging to the region of interest (ROI), considered for the computation of the 95th percentile of MPS, MPSR, MPSXSR, and MSS. **(C)** Maximum principal strain distribution on a parasagittal cross section of the brain when the probe reaches maximum depth. The image is taken from one of the FE CCI simulations used to train the ANN. The input parameters were: a hemispherical probe (diameter = 15 mm), velocity of indentation = 1.8 m/s, depth of indentation = 5.4 mm, angle of inclination = 3°, DAM-probe friction coefficient = 0.24, and thickness of the meninges = 0.655 mm. The highest MPS values are concentrated around the impact point, within the ROI.

The skull was generated by extending the DAM geometry outwards. A circular hole (diameter = 23 mm) on the superior part of the skull was created in 3-Matic to mimic the unilateral craniotomy and to virtually expose the dura mater. Here, the discrete rigid skull surface is acting as a geometrical boundary to constrain unrealistic deformations of the DAM and the brain, rather than being a strict anatomical representation of the porcine cranial bones (Figure 2).

All the porcine geometrical parts were imported into ANSA (BETA CAE Systems) for mesh generation, which was performed using quality control criteria that included an aspect ratio <3, skewness >0.7, Jacobian >0.7, minimum angle for triangles and tetrahedrons >30°, and maximum angle for triangles and tetrahedrons <120°. The meshes of the brain and DAM were refined in the vicinity of the impact point of the probe. A convergence analysis was carried out on the brain mesh to ensure stability and sufficient resolution of the mesh while minimizing the computational runtime. The optimal brain mesh resulting from the convergence analysis findings counted 203,167 quadratic tetrahedral elements of type C3D10M from the Abaqus/Explicit v.2019 (Dassault Systémes) library. This was preferred over the regular second-order tetrahedrons of type C3D10 due to its superior performance in minimizing volumetric locking for nearly incompressible materials. The skull mesh consists of 3,090 linear triangular discrete rigid elements, while the DAM consists of 20,590 linear triangular shell elements (type S3R) with thickness that varies between 0.413 and 1.058 mm (see *Input and Output Parameters of the FE Model*) to cover the range of values reported by Vanmol (2017) and Walsh et al. (2018). Linear triangular discrete rigid elements were used to mesh the probe, with the number of elements varying between 498 and 5,324 depending on the diameter size.

Both the brain and the DAM were considered to be homogeneous and isotropic materials, using neo-Hookean–based quasi-linear viscoelastic models. The material properties were retrieved from MacManus et al. (2017), who characterized the mechanical behavior of the porcine brain and the DAM under large deformation by means of dynamic indentation *in situ*. Specifically, the viscoelastic parameters were obtained by fitting the results of an inverse finite element model of the indentation experiments with simulated loading conditions representative for the CCI.

The presence of CSF was included by using the fluid-filled cavity definition available in Abaqus to model liquid-filled structures enclosed by surfaces. The density and bulk modulus of water were used to approximate the CSF (Kleiven, 2007). Table 2 provides a summary of all the material parameters used in the FE model of the porcine brain.

**TABLE 2:** Material properties used in the FE model of the porcine brain FE.

Part	Density	Material model	Material parameters
Brain	1,060 kg/m^3^	Neo-Hookean–based quasi-linear viscoelastic	μ = 6.97 kPa
			ν = 0.49995
		MacManus et al. (2017)	$g_{1}$ = 0.451 $g_{2}$ = 0.301
			$\tau_{1}$ = 0.021 s $\tau_{2}$ = 0.199 s
Dura-arachnoid mater (DAM)	1,000 kg/m^3^	Neo-Hookean–based quasi-linear viscoelastic	μ = 19.10 kPa
			ν = 0.4
		MacManus et al. (2017)	$g_{1}$ = 0.568 $g_{2}$ = 0.240
			$\tau_{1}$ = 0.034 s $\tau_{2}$ = 0.336 s
Cerebrospinal fluid (CSF)	1,000 kg/m^3^	Homogeneous fluid–filled cavity	*K* = 2.1 GPa

*μ*, shear modulus; *ν*, Poisson’s ratio; $g_{1}$ and $g_{2}$ , first and second term of the relaxation function of the Prony series that describes the viscoelastic behavior; $\tau_{1}$ and $\tau_{2}$ , first and second term of the time constant of the Prony series that describes the viscoelastic behavior; *K*, bulk modulus.

The “general contact” definition was used to govern the interactions between parts, with a penalty friction formulation to model the interaction between the probe and the outer surface of the DAM. The friction coefficient varied between 0.07 and 0.26 (see *Input and Output Parameters of the FE Model*) or the range of values reported by Rashid et al. (2012), who estimated the friction between the porcine brain and metal platens with unconfined compression tests. Here, we assumed that the porcine brain and DAM have the same frictional behavior against metal. Only one hemisphere was considered in the FE model; therefore, we constrained the out-of-sagittal plane linear and rotational motion of the medial side of the DAM to mimic the presence and mechanical role of the falx (Walsh et al., 2021). In a similar way, given the distance from the area of influence to the impacting probe, we simplified the presence of the diencephalon, the brainstem, and the cerebellum by constraining any translation of the inferior part of the DAM. The simulated time was varied as a function of the assigned probe’s displacement-time profile to include 50 ms of dwell time after the probe reached the maximum indentation depth. The simulations were run using the explicit solver of Abaqus v. 2019, which accounted for geometric nonlinearities throughout the whole computation. Element mass scaling was introduced if the stable time increment decreased below 1.2 × 10^−6^ s. We verified that the total percent change in mass of the model resulting from mass scaling was <4% for all simulations.

With the aim of collecting data to validate the assumptions made in the FE model, we performed an additional test *in vivo* on a pig (from an unrelated experiment) to measure the reaction force exerted by the brain on the probe during a CCI, since De Kegel et al. (2021) reported no quantitative assessment of the mechanical response of the pigs’ brains to the impacts. To this extent, a dynamic load cell (PCB 208B, load capacity = ±45 N, resolution = 0.0009 N) was mounted on the CCI device using a custom-made nylon support. The hemispherical probe was connected to the load cell by means of a lightweight, aluminum threaded support. The pig underwent the same testing protocol as described in De Kegel et al. (2021), with the same velocity and depth of indentation of case III (Table 1), that is, 1.8 m/s and 9.1 mm. During the impact, the force in the direction of the axis of the probe was measured with a sampling rate of 5,000 Hz. Subsequently, the CCI was simulated with the porcine brain FE model, and the force exerted by the brain on the probe in the direction of its axis was computed. The computational and experimental force-time signals were compared and their correlation was quantified using the software CORA (Correlation and Analysis, Version 3.6.1) (Gehre et al., 2009) to verify that the brain impact mechanics was correctly modeled. The CORA software combines two independent rating techniques (the cross-correlation and the corridor method) to express the level of similarity between the experimental and computational curves with a score between 0 and 1, with 1 meaning perfect correlation. The two ratings had equal weight on the overall rating (G1 = G2 = 0.5). Regarding the cross-correlation–based score, we set equal weights (= 1/3) to amplitude, shape, and phase errors. A linear transition between ratings was chosen ( $k_{V}=k_{P}=\;k_{G}=1)$ .

### Input and Output Parameters of the FE Model

Seven parameters of the FE model were considered for the uncertainty and sensitivity analyses due to the relatively large spread of values reported in the literature. The list included the probe’s diameter and shape (hemispherical or cylindrical), the velocity and the depth of indentation, the friction between the DAM and the probe, the inclination angle between the axis of the probe and the normal of the DAM surface, and the thickness of the DAM. Each of these input parameters varied between a lower and upper bound with specific statistical distributions (Table 3). The lower and upper bounds for the probe geometrical and kinematics parameters (i.e., diameter, shape, angle of inclination, and velocity and depth of indentation) were chosen to comprise the values reported in the literature for CCI tests on pig brain (Madsen and Reske-Nielsen, 1987; Duhaime et al., 2000; Alessandri et al., 2003; Manley et al., 2006; Meissner et al., 2011; Sindelar et al., 2017; De Kegel et al., 2021). For the friction between the probe and the DAM, the coefficients obtained experimentally by Rashid et al. (2012) were used. Finally, the measures on the porcine meninges performed by Vanmol (2017) and Walsh et al. (2018) were used to determine the lower and upper bound of the DAM thickness.

**TABLE 3:** Range of values and statistical distribution of the input parameters of the porcine brain FE model considered for the uncertainty and sensitivity analyses. For the DAM-probe friction and DAM thickness parameters normal distributions are used, since their values are measured experimentally. The parameters that are selected upon designing the CCI experiments have either a discrete or uniform distribution.

Input parameter	Lower bound	Upper bound	Statistical Distribution
Diameter probe	5 mm	20 mm	Discrete
Shape of the tip of the probe	0 (=cylindrical)	1 (=hemispherical)	Discrete
Velocity of indentation	0.4 m/s	4 m/s	Uniform
Depth of indentation	1.1 mm	12.6 mm	Uniform
Inclination of the probe	−15°	15°	Uniform
DAM-probe friction	0.07	0.26	Normal
Thickness DAM	0.413 mm	1.058 mm	Normal

The FE porcine brain model was used to compute the mechanical response of the brain to the CCI. We considered four different tissue-level injury metrics: the logarithmic maximum principal strain (MPS), the logarithmic maximum principal strain rate (MPSR), the product of logarithmic maximum principal strain and strain rate (MPSXSR), and the logarithmic maximum shear strain (MSS). As estimators of CC, we computed the 95th percentile of the peak MPS, MPSR, MPSXSR, and MSS of the brain elements belonging to the region of interest (ROI) in the brain that is directly affected by the CCI, namely, where the primary focal traumatic brain injury can arise (Figure 2). The elements were considered as a part of the ROI if their centroid was within a 40 mm distance from the impact point on the brain surface. We did not consider the 100th percentile of the peaks in order to discard any unrealistically high values due to computational artifacts (Panzer et al., 2012).

### Machine Learning–Based Surrogate Model

To perform the uncertainty quantification in a computationally efficient way, we developed a surrogate of the FE porcine brain model. An artificial neural network (ANN) was employed as the surrogate modeling technique, which was implemented in TensorFlow 2.0.2 (Abadi et al., 2016). The ANN algorithm was adapted from Bartsoen et al. (2021), who demonstrated the superior accuracy performance of ANN with respect to other probabilistic modeling techniques based on the response surface method, such as 2nd order polynomial, Gaussian process regression, or support vector regression (Laz and Browne, 2010). To train and validate the ANN, we generated a total of n samples, where each sample consisted of a set of seven input values (one per input parameter) selected with the Sobol sequence among the ranges indicated in Table 3, and four outputs (the 95th percentile of MPS, MPSR, MPSXSR, and MSS) computed with the FE porcine brain model. The amount of training and validation samples were 0.9 n and 0.1 n , respectively.

The architecture of the network consisted of fully connected layers (7:256:128:64:32:16:4) with the Softplus activation function a(x)=ln(1+ exp(x)) , where x is the input to a neuron. The output layer had the ReLU activation function $a\left(x\right)=x^{+}=\text{max}\left(0,\;x\right)$ to exclude negative outputs. As our loss function, we used the Huber loss function, as given below:

$$\{\begin{array}{cc} \frac{1}{2}e^{2} & if\;\left|e\right|\leq d\\ \frac{1}{2}\;d^{2}+d\left(\left|e\right|-d\right) & if\;\left|e\right|>d \end{array},$$ (1)

where e represents the residuals, that is, the difference between the observed (y) and predicted values $\left(\hat{y}\right)$ : $e=y-\hat{y}$ , while d is a parameter set equal to 0.1. The Huber loss computed using Eq. 1 has a similar behavior as the mean squared error (MSE) for small errors but the mean absolute error (MAE) for large errors so that it is less affected by outliers. The network was trained using an Adam optimizer with a learning rate = 0.001 that decays upon convergence of the loss. To prevent overtraining, L2 kernel regularization was applied. The ANN accuracy was further improved with ensemble averaging, in which we considered a weighted average of six networks trained on the same training and validation data. The weights were optimized by minimization of the validation MSE. The normalized mean absolute error (nMAE), normalized root mean squared error (nRMSE), and 95% absolute error (95% AE) were evaluated. The nMAE and nRMSE were computed after normalizing the output range to [0;1], while the 95% AE gives the 95th percentile of the AE. The optimal number of samples used to train and validate the ANN was selected after carrying out a 5-fold cross-validation. The ANN performance was evaluated considering the nMAE, nRMSE, and 95% AE on the validation and training samples for each of the four outputs, considering a total of 10, 20, 40, and 80 samples. The minimum number of samples that yielded nMAE, nRMSE, and 95% AE < 4% was 80 (training samples = 72, validation samples = 8).

With the ML surrogate model, we achieved a considerable increase in computational efficiency compared to a typical FE model simulation. The average computational time to simulate a CCI with the FE porcine brain model was approximately 2 h when parallelizing the execution between 144 domains (Xeon Gold 6140, CPUs at 2.3 GHz) using the high-performance computing centrum (VSC—Flemish Supercomputer Center). On the contrary, generating 80 FE samples took ∼160 h, while the training of the ANN was completed in about 7 min. Predicting the values of the CC injury estimators with the trained ANN was immediate (<0.01s); therefore, the advantage of utilizing a surrogate model for uncertainty and sensitivity analyses is clear, especially when considering the large number of simulations necessary for the sensitivity analysis (see *Sensitivity Analysis*).

### Sensitivity Analysis

Once the ANN was trained and tested, we utilized the surrogate model as basis for the Monte Carlo simulations because of its very low computational cost in generating a large number of samples (Laz and Browne, 2010). Knowing the statistical distributions of the input parameters (Table 3), we performed the uncertainty analysis using the MCS with 10,000 samples. In each MCS, one parameter was kept constant and equal to a value inside the bounds according to its statistical distribution. In particular, the shape had two possible levels (0 = cylindrical probe and 1 = hemispherical probe), the diameter of the probe had four (5–10–15–20 mm), and all the other parameters had ten each (ten values equally spaced between the lower and the upper bound, shown in Table 3). Therefore, we repeated the MCS 56 times in order to assess the effect of each individual input on the injury metrics.

To rank the importance of the influence of the input uncertainty on the output distribution, we computed two global indicators using the open source Python’s library SALib. The first one is the δ moment-independent measure (Borgonovo, 2007)

$$\delta_{i}=\;\frac{1}{2}\;E_{X_{i}}\left[s\left(X_{i}\right)\right],$$

where $\delta_{i}$ is the moment-independent indicator of the sensitivity of the output, Y , to the input parameter $X_{i}$ , and $E_{X_{i}}\left[s\left(X_{i}\right)\right]$ represents the expected shift in the distribution of Y provoked by $X_{i}$ .

The second indicator considered here is the first-order sensitivity index S1 (Sobol, 2001),

$$S1_{i}=\;\frac{V_{i}}{V\left[Y\right]},$$

where $S1_{i}$ is the first-order sensitivity index specific to the input parameter $X_{i}$ , which represents the expected reduction in the variance of the output V[Y] in a hypothetical case in which the uncertainty relative to the input parameter, $X_{i}$ , would be excluded.

One common mathematical property for both indicators is that given an input parameter, $X_{i}$ , for a fixed output, Y , the closer $\delta_{i}$ or $S1_{i}$ are to 1, the more important the parameter $X_{i}$ is for Y . In the extreme case, if $\delta_{i}\text{ or }S1_{i}$ = 1, then Y only depends on $X_{i}$ ; conversely if $\delta_{i}\text{ or }S1_{i}$ = 0, then $X_{i}$ has no influence on Y . The seven $\delta_{i}$ ’s and seven $S1_{i}$ ’s were estimated after generating 10,000 sets of input parameters by means of Latin Hypercube sampling, which were used in the ML surrogate model to predict the 95th percentile of MPS, MPSR, MPSXSR, and MSS.

### Performance Evaluation of the CC Injury Metrics

The case-specific 95th percentile MPS, MPSR, MPSXSR, and MSS were predicted by the trained ANN using the known experimental parameters of each CCI test as input. For all the CCI cases we used diameter of the probe = 10 mm, inclination of the probe = 0°, and shape of the tip = 1 (hemispherical). We assumed friction of the DAM-probe = 0.163 mm and thickness of the DAM = 0.736 mm, that is, the mean values of the corresponding normal distributions (Table 3). The velocity and depth of indentation were chosen depending on the values specific for each test (Table 1). In order to use binary values to express the presence or absence of visible disruption of the cortical layer (Table 1), we used the dummy variable “1” when CC was confirmed and “0” when no injury was detected.

A Wilcoxon Rank-Sum test (significance level = 0.05) was carried out to assess the effectiveness of each injury estimator in distinguishing between CCI cases with cortical damage and without cortical damage.

The injury risk curves that express the likelihood of sustaining CC depending on the different FE-derived injury predictors were obtained by performing univariate logistic regression, a technique that has been broadly used to determine injury tolerances based on experimental data and predict the outcome of TBI (Shreiber et al., 1997; Takhounts et al., 2003; Kleiven, 2007; Steyerberg et al., 2008; Cai et al., 2018; Anderson et al., 2020; Hajiaghamemar et al., 2020). We selected a logistic regression classifying method due to the binary nature (CC or no CC) of the outcome of the CCI data, obtained by means of the post-injury MR scans and histology assessments (De Kegel et al., 2021). In the logistic regression model the probability p of sustaining CC as function of an injury predictor X is described by the relationship

$$p\left(X\right)=\;\frac{1}{1+\text{exp}\left(-b_{0}+\;b_{1}\cdot X\right)},$$

where $b_{0}$ and $b_{1}$ are the regression coefficients.

The accuracy of the injury metric-specific prediction of CC likelihood was evaluated using a leave-one-out cross-validation (LOOCV). We selected a LOOCV framework because of its low bias and the limited size of our dataset (14 samples) (Beleites et al., 2005; Cai et al., 2018; Anderson et al., 2020). In order to perform an objective comparison between the performances of the univariate logistic regression classifiers, we computed the LOOCV accuracy, sensitivity, and specificity. We also reported the AUC, that is, the area under the receiver operating characteristic (ROC) curve, for both the training and the testing datasets, similar to Cai et al. (2018) and Anderson et al. (2020). In particular, the metric-specific AUC value for the training dataset was calculated as the average of the AUCs of 14 independent injury predictions and each one obtained considering 13 simulated CCI cases, conforming to the LOOCV framework. The metric-specific AUC value for the testing dataset was determined taking into account the 14 independent injury predictions. The implementation of the LOOCV framework and the performance evaluation of the injury estimators were carried out using Python’s machine learning library scikit-learn.

## Results

**FIGURE 3:**
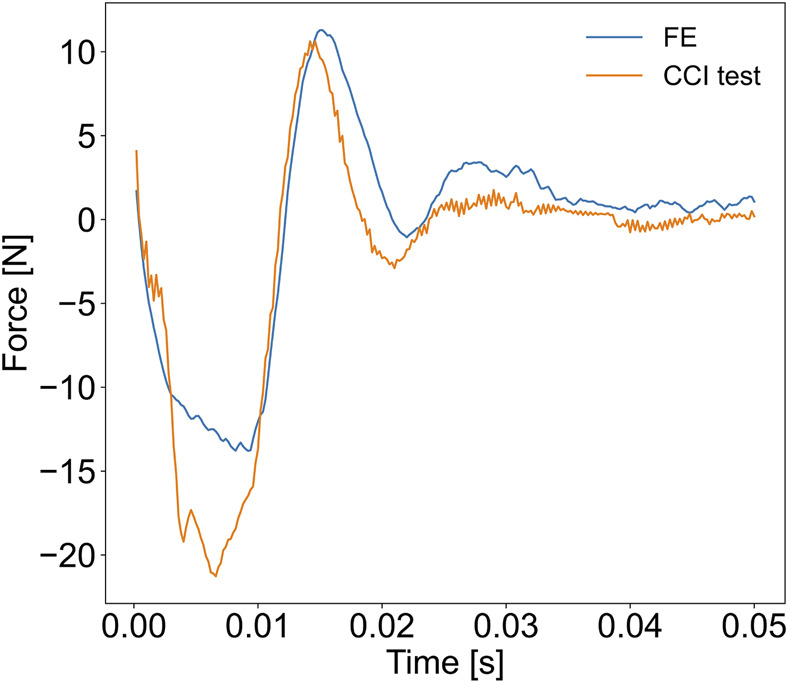
Results of the validation of the FE porcine brain model. The reaction force exerted by the impacted brain on the probe during the CCI was measured experimentally and compared with the force-time history simulated with the FE model.

To validate the FE model of the porcine brain undergoing CCI, we calculated the CORA score by comparing the experimentally measured and the simulated force-time curves of the probe. The overall CORA rating was equal to 0.827 (the cross-correlation method = 0.880 and the corridor method = 0.773), which corresponds with the “good biofidelity” category. Therefore, the FE model yielded a sufficiently accurate prediction of the localized force-time response of the pig’s brain to CCI (Figure 3).

**FIGURE 4:**
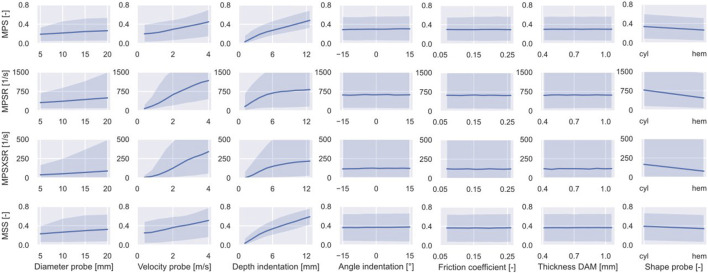
Outcome of the MCS performed on the surrogate of the porcine brain FE model. The variability of the CC injury metrics (i.e., 95th percentile of MPS, MPSR, MPSXSR, and MSS in the ROI) is shown as a function of the input parameters. The solid line represents the median while the shaded area delimits the 90% confidence interval.

The 95th percentiles of the MPS, MPSR, MPSXSR, and MSS of 10,000 simulations were predicted using the ML-based surrogate of the porcine brain’s FE model. The results of the MCS are shown in Figure 4, which displays the effect (indicated by the slope of the curves representing the median response) that each individual input parameter has on the predicted injury metrics, as well as on their variability. Interestingly, the angle of inclination, the DAM-probe friction, and the thickness of the DAM have minimal or no influence on the four injury metrics. The difference in shape of the probe’s tip has little effect on MSS and MPS, while it produces more pronounced discrepancies in terms of MPSR and MPSXSR. In particular, a hemispherical probe yields both lower strain and strain rates. The median of all four injury metrics increases quasi-linearly with the diameter of the probe; however, their variability (i.e., the 95% confidence interval bound) increases nonlinearly when considering larger diameters. Although all injury metrics increase with the velocity of indentation, the quasi-linear relationship observed with MPS and MSS is not apparent for MPSR and MPSXSR where large variability for higher speeds is visible. Large variability at higher values and nonlinearity of the median are also noticeable for MPSR and MPSXSR as a function of the depth of indentation. MPS and MSS exhibit quasi-linear and monotonically increasing relations with the depth of indentation. The variability of MPS and MSS as a function of the depth of indentation is the lowest of all, suggesting that the depth of indentation has a prominent role in the definition of the brain strain field.

**FIGURE 5:**
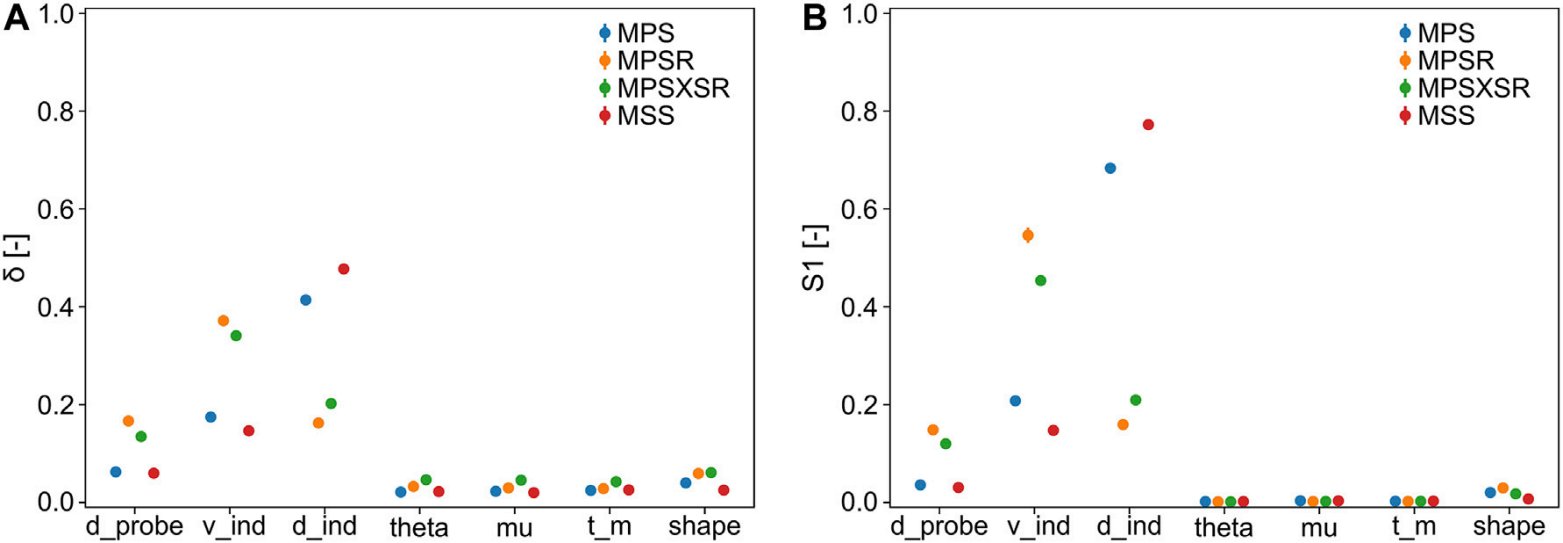
Sensitivity indicators (with 95% confidence interval) of the injury metrics with respect to the input parameters (d_probe = diameter of the probe, v_ind = velocity of indentation, d_ind = depth of indentation, theta = inclination of the probe, mu = DAM-probe friction, t_m = thickness DAM, and shape = shape of the tip of the probe). **(A)** δ moment-independent measure; **(B)** S1 first-order sensitivity index.

The results of the sensitivity analysis with 10,000 samples are displayed in Figure 5. Both sensitivity indicators show a very similar trend in terms of ranking the importance of the input parameters despite showing different absolute values. In particular, the most important parameter for MPS and MSS is the depth of indentation (MPS: δ = 0.414 ± 0.008, S1 = 0.683 ± 0.008; MSS: δ = 0.477 ± 0.007, S1 = 0.772 ± 0.004), while the parameter with the biggest influence on MPSR and MPSXSR is the velocity of impact (MPSR: δ = 0.372 ± 0.008, S1 = 0.546 ± 0.015; MPSXSR: δ = 0.341 ± 0.007, S1 = 0.453 ± 0.011). On the contrary, the angle of inclination, the DAM-probe friction, and the thickness of the DAM exhibited little or no influence on any of the four injury metrics, which was demonstrated by both δ (<0.05 for all injury metrics) and S1 (<0.005 for all injury metrics). The diameter of the probe ranked as the third most influential parameter on all injury metrics (MPS: δ 0.063 ± 0.003, S1 = 0.036 ± 0.007; MPSR: δ = 0.167 ± 0.004, S1 = 0.148 ± 0.010; MPSXSR: δ = 0.135 ± 0.008, S1 = 0.120 ± 0.011; MSS: *δ* = 0.060 ± 0.003, S1 = 0.031 ± 0.007). The shape of the probe exhibited limited influence on the outcome of the simulations, with slightly higher δ in case of MPSR and MPSXSR (0.059 ± 0.005 and 0.061 ± 0.003, respectively) and slightly higher S1 when considering MPSR (= 0.030 ± 0.005). The complete table with δ moment-independent measures and first order sensitivity indices S1 of the injury metrics as a function of the input variables is available in the Supplementary Material.

**FIGURE 6:**
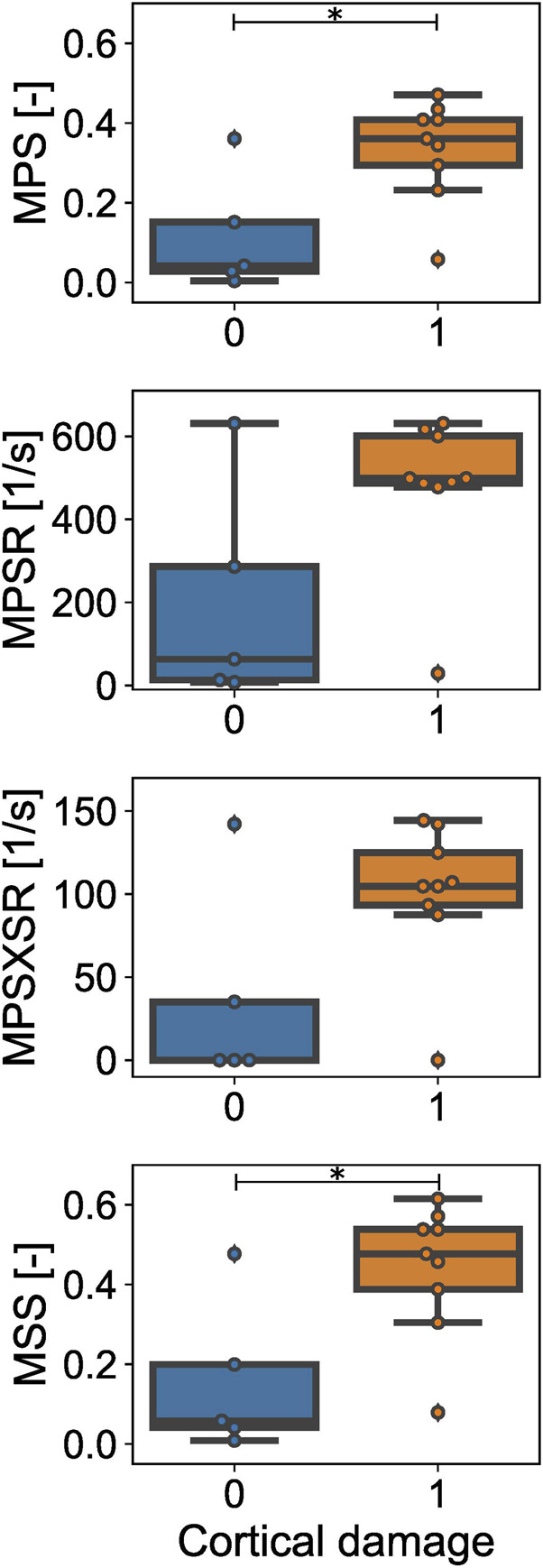
Estimated values of the injury metrics for the CCI injury (orange) *vs.* no injury (blue) cases. Maximum principal strain (MPS) and maximum shear strain (MSS) are different for cases with observed cortical damage *vs.* no damage. No statistical difference is observed in terms of maximum principal strain rate (MPSR) and maximum principal strain x strain rate (MPSXSR) between injury and no injury cases. * = *p*-value < 0.05.

The effectiveness of each scalar injury metric in separating between cases with CC and without CC based on the magnitude of the estimator was assessed *via* the Wilcoxon Rank-Sum test. The statistical analysis (Figure 6) confirmed that the 95th percentiles of MPS and MSS were significantly different between the simulated cortical damage and no damage cases (*p*-value = 0.0234 in both cases). Conversely, within the considered dataset, the 95th percentile of MPSR and MPSXSR of the injury and no injury groups were not significantly different (*p*-values = 0.110 and 0.083, respectively).

The LOOCV procedure was implemented to test the robustness and compare the performance of the metric-specific univariate logistic regressions. Within the LOOCV framework, the accuracy, sensitivity, specificity, and AUC on the testing dataset scores were equivalent for all the FE-based injury predictors (Table 4). However, the AUC score calculated on the training datasets revealed that MPS and MSS (both average AUC = 0.878) outperformed MPSR (0.767) and MPSXSR (0.789). The ROC curves for both the testing and training datasets are available in the Supplementary Material.

**TABLE 4:** Summary of the performance scores of the four different injury predictors. The scores were obtained using the leave-one-out cross-validation framework. The AUC-training values are presented as the average (standard deviation) of the testing datasets. The best and worst AUC-training measures are also reported.

Injury metric	Accuracy	Sensitivity	Specificity	AUC-testing	AUC-training	Best AUC-Training	Worst AUC-Training
95th MPS	0.784	0.8	0.75	0.711	0.878 (0.032)	0.972	0.847
95th MPSR	0.784	0.8	0.75	0.711	0.767 (0.055)	0.944	0.708
95th MPSXSR	0.784	0.8	0.75	0.711	0.789 (0.049)	0.944	0.736
95th MSS	0.784	0.8	0.75	0.711	0.878 (0.032)	0.972	0.847

**FIGURE 7:**
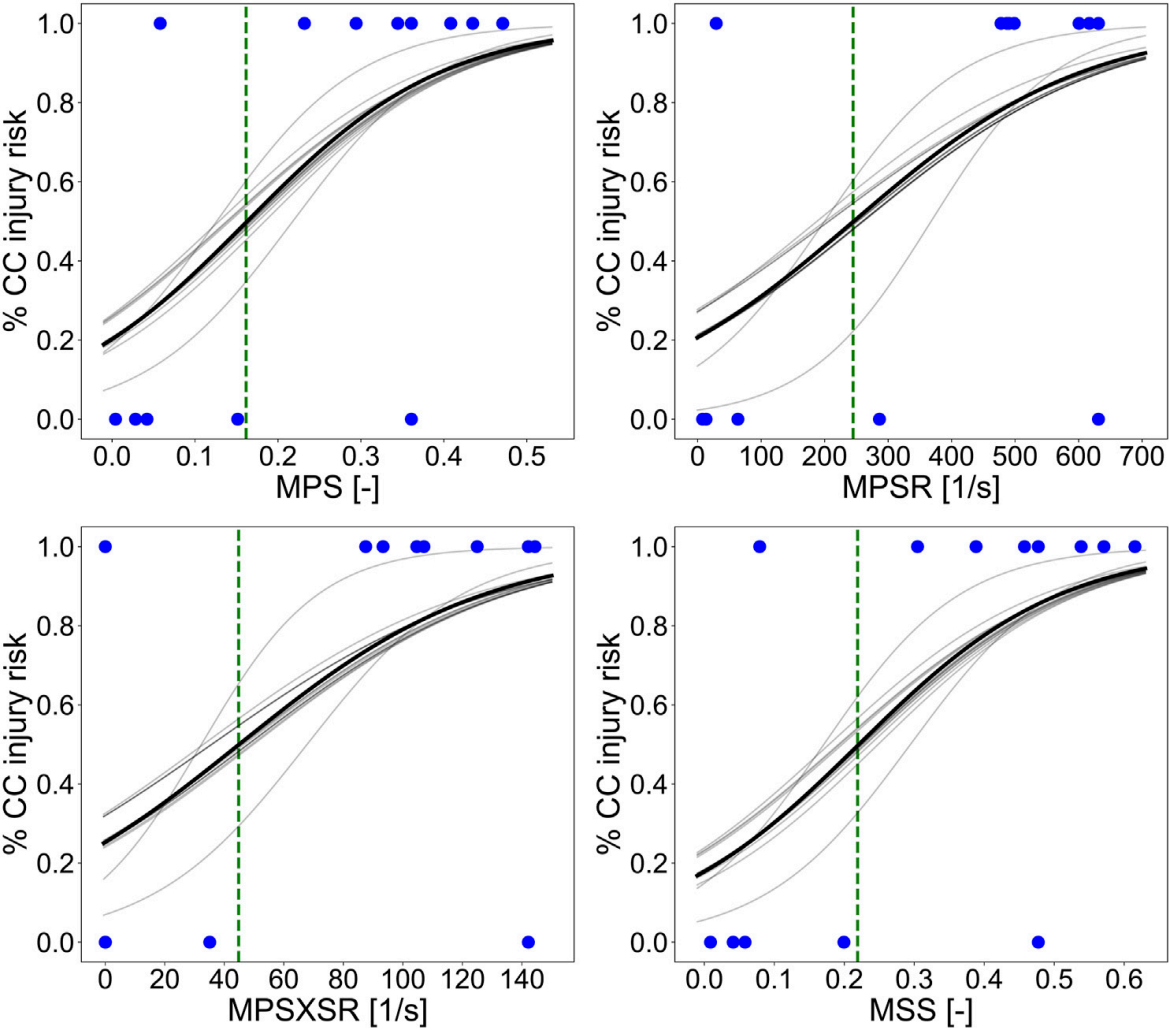
Injury risk curves representing the probability of sustaining CC as a function of the 95th percentile of MPS, MPSR, MPSXSR, and MSS predicted with the porcine brain ML surrogate. The solid black line represents the logistic regression curve performed on the whole dataset. The light gray lines represent the logistic regression curves of each training dataset of the leave-one-out cross-validation procedure. The green vertical dashed lines indicate the thresholds corresponding to the 50% chance of CC (MPS = 0.16, MPSR = 245 s^−1^, MPSXSR = 45 s^−1^, and MSS = 0.22), while the blue dots represent the experimental data (cortical damage = 1 and no damage = 0) collected by means of the MR and histology assessments post-CCI.

Figure 7 illustrates the injury risk curves representing the probability of sustaining CC as a function of the 95th percentile of MPS, MPSR, MPSXSR, and MSS predicted with the porcine brain ML surrogate model and based on the *in vivo* CCI data. We determined the metric-specific injury thresholds by computing the value of the predictors that corresponds to a 50% probability of sustaining CC, estimated using the univariate logistic regression curve of the whole dataset. The obtained thresholds were MPS = 0.16, MPSR = 245 s^−1^, MPSXSR = 45 s^−1^, and MSS = 0.22.

## Discussion

### FE Porcine Brain Model

The FE model simulated the intracranial response of the porcine brain undergoing CCI to explore the relationship between the deformation and rate of deformation of the cerebral tissue and the risk of sustaining CC. Besides the reduced simulation runtime resulting from using a simplified geometry, the rationale behind the development of a single brain hemisphere model was the focal nature of CC induced by CCI tests, which are designed to damage only the ipsilateral side of impact (Alessandri et al., 2003; Elliott et al., 2008). Contrary to other porcine head FE models that cope with loading conditions involving the kinematics of the whole head (Coats et al., 2012; Yates and Untaroiu, 2016; Hajiaghamemar and Margulies, 2021), the current model is conceived to simulate localized impacts only, therefore the geometrical simplification adopted is sufficient. The validation carried out by comparing the impact forces supported the suitability of the FE model and its boundary conditions. However, no experimental measurement of the brain tissue deformation was available for further validation, which, therefore, represents a limitation. *In vivo* measurements of the deformation of the brain tissue during CCI are practically very challenging, given the very limited visibility of the cortex and accessibility between the probe and the craniotomy burr hole. Future validation efforts could consider measuring dynamic cortical deformation *in vivo* by applying vacuum pulses on the exposed parenchyma, as reported by Schreiber et al. (1997) and Mao et al. (2006) using rat brains. The stability of the model was ensured for all the simulations, where no excessive distortion of the deformable parts (i.e., brain and DAM) was observed.

The assumptions of homogeneity and isotropy of both the brain-pia mater and DAM complexes could represent a matter of debate for the present FE model. Indeed, various studies have reported that porcine gray and white matter might display different stiffness, although these differences seem to depend on the testing conditions (Prange and Margulies, 2002; Gefen and Margulies, 2004; van Dommelen et al., 2010; Elkin et al., 2011; Kaster et al., 2011; Li Z. et al., 2020). Moreover, integrating the pia mater with the underlying brain tissue might underestimate the protective role of the innermost meningeal layer, which exhibits a higher elastic modulus than the brain (Li Y. et al., 2020; Walsh et al., 2021). While it is generally agreed upon that gray matter shows an isotropic behavior, it has been reported that white matter exhibits a degree of anisotropy in regions with significant axonal fiber alignment such as the corpus callosum (Prange and Margulies, 2002; van Dommelen et al., 2010; Feng et al., 2017). On the other hand, the homogeneity and isotropy of the mechanical behavior of the cerebral dura mater, in spite of the presence of collagen fibers, has been confirmed in different studies (De Kegel et al., 2018; Pierrat et al., 2020). Nevertheless, there is no constitutive law capable of capturing the mechanical behavior of the cerebral and meningeal tissues under any arbitrary loading condition, which necessitates that the selected material models be calibrated under testing conditions similar to the problem considered (de Rooij and Kuhl, 2016). Therefore, the neo-Hookean quasi-linear viscoelastic models for the brain-pia mater and DAM complexes obtained by MacManus et al. (2017) were a reasonable choice, as they were calibrated with dynamic indentations of the brain surface.

Several approaches have been adopted in the literature to model the presence of CSF in FE head models, such as the use of linear elastic solid elements for the CSF (Kleiven, 2007; Coats et al., 2012; Trotta et al., 2020), or fluid-structure interaction (Zhou et al., 2019). Other studies incorporated the cerebral vasculature in the FE head models and found that it reduces the brain strains resulting from blast exposures (Unnikrishnan et al., 2019) and head collisions (Zhao and Ji, 2020b). Coats et al. (2012) explored the possibility of modeling the brain-skull interface by replacing the pia-arachnoid mater complex, CSF, and blood vasculature with spring connector elements with assigned stiffness of cortical veins. This suggests that the vascular structure is an important constituent that is affected by nonimpact large head rotations. These rotations, however, do not reflect the mechanical loading scenario of a CCI. Therefore, we hypothesize that the fluid-filled cavity approach used here is sufficient to model the presence of an incompressible material between the impacted DAM and the brain. Future developments of the porcine brain FE model should explore the load-bearing behavior of the structural interaction between the CSF and the cortical vasculature during direct impacts.

### Machine Learning Model

A surrogate of the porcine brain FE model was fitted using an artificial neural network with the aim of performing uncertainty and sensitivity analyses with reduced simulation runtime and computing resources. The trained and tested ANN was able to predict the 95th percentile MPS, MPSR, MPSXSR, and MSS of the porcine brain undergoing CCI in <0.01s using a low-end laptop, contrarily to the ∼2 h runtime required to simulate a single impact with the “traditional” FE porcine brain model using a high-performance computing platform. Considering the large number of samples required by the MCS (56 times 10,000), the computational time saved by performing the uncertainty analysis on the ML surrogate model is evident.

We reached the desired accuracy for uncertainty quantification by using only 80 samples in the training and testing process for the ANN. Most machine learning–based models would require more samples to be able to achieve errors <4%; however, since only three out of the seven input parameters were proved to be highly influential, this relatively low number of training and validation samples is plausible.

The fully connected seven-layers ANN architecture used in this study is relatively simple and only considers a one-dimensional array as input. There exist more complex deep learning–based surrogates of FE head models in the literature, such as convolutional neural networks (CNN) (Wu et al., 2019; Ghazi et al., 2021). Improving the outcomes from Wu et al. (2019), Ghazi et al. (2021) combined the head impact rotational velocity and acceleration temporal profiles into a two-dimensional input to train a CNN architecture composed of convolutional, pooling, flattening, and fully connected layers. The CNN was trained to predict the MPS distribution across the whole brain of the anisotropic Worcester Head Injury Model (Zhao and Ji, 2020a). With a conceptually similar workflow, Zhan et al. (2021) utilized features describing the characteristics of rotational kinematics of football and mixed martial arts’ head impacts as input of a five-layer deep neural network to predict the MPS map of the brain elements of the KTH head model (Ho and Kleiven, 2007).

The remarkable advantage of these approaches is the preservation of the information about the nonlinear impact–whole brain deformation relationship, which is otherwise inevitably lost when considering discrete inputs and single-value metrics outputs as in our study. Nevertheless, the focal nature of the cortical damage induced by the CCI reduces the relevance of predicting the brain mechanical response outside the ROI that is directly affected by the impacting probe. The ANN was, therefore, sufficient to achieve the goal of estimating the effect of the experimental and modeling parameters on the localized strain and strain rate of the impacted pig brain. Future studies should investigate the use of more sophisticated deep learning networks that include the nonlinear temporal profile of input parameters such as depth and velocity of indentation, which, due to the dynamic nature of the brain tissue properties, might influence the predicted intracranial mechanical response.

### Sensitivity to the CCI Model Parameters

The uncertainty and sensitivity analyses demonstrated that the selected input parameters have different degrees of influence on the deformation and rate of deformation of the porcine cerebral tissue undergoing CCI. Both sensitivity indices, S1 and δ , highlighted the predominant effect of the depth of indentation on strain-based injury metrics. The crucial effect that increasing the depth of indentation has in terms of both functional responses and tissue structural injury pattern is well reported in the literature of CCI on rodent (Goodman et al., 1994; Saatman et al., 2006; Elliott et al., 2008; Mao et al., 2010a) and pig brains (Manley et al., 2006; Baker et al., 2019). The evaluation of the uncertainty propagation showed that by eliminating the uncertainty on the depth of indentation in a CCI experiment, one could dramatically reduce the variability of MPS and MSS. This demonstrates that this input parameter should be carefully selected and measured when performing a CCI experiment. Interestingly, MPSR and MPSXSR seem to reach a plateau for depths of indentation approaching 12 mm, which suggests that it may not be crucial for the impactor to reach depths larger than 12 mm in order to produce further damage. This is in agreement with Manley et al. (2006) and Baker et al. (2019), who both observed that CCI carried out on pig brain with a depth of indentation greater or equal than 12 mm produces largely extended areas with neuroparenchymal damage and loss and intraparenchymal hematoma, which overshadows the mere cortical contusion.

The other critical parameter is the velocity of impact, which unsurprisingly ranked first in order of importance with the strain rate injury metrics, MPSR and MPSXSR. This is a logical consequence of the viscoelastic nature of the brain tissue. The strain rate dependence introduced by the brain tissue viscoelastic material model could also explain why large asymmetry in the uncertainty distribution are observed for MPSR and MPSXSR, especially for higher values of velocity and depth of indentation. Indeed, it is known that brain tissue shows more viscous behavior and its shear modulus exhibits a more rapid decay with increasing strain rates (Qian et al., 2018).

The effect of the interaction between the depth and velocity of indentation was not investigated in our study. However, CCI experiments on pig brain by Baker et al. (2019) showed that the interaction between these two parameters influence the cortical lesion size and the observable functional deficit. The combined effect of depth and velocity of indentation on brain tissue deformation and deformation rate should be object of future research.

The trends of the median of all four injury predictors observable in Figure 4 reveal that the geometrical properties of the probe influence the brain’s response to the CCI, although less than the depth or the velocity of impact. Unsurprisingly, a larger diameter of the probe results into larger strains and strain rates. Moreover, hemispherical probes are associated with lower MPS, MPSR, MPSXSR, and MSS than cylindrical flat tips, which is explained by the higher concentration of stresses due to smaller radii. This is confirmed by the CCI experiments on mice by Pleasant et al. (2011), who observed a greater acute cortical hemorrhage and neural loss with a flat probe when compared to a hemispherical one.

The uncertainty of the angle of indentation, the friction coefficient between the DAM and the probe, and the thickness of the DAM show no meaningful effect on the propagation of the uncertainties of all the injury metrics. This demonstrates that committing an error in positioning the axis of the probe with an angle relative to the surface of the brain parenchyma in the range [−15°; 15°] is tolerable in terms of achieving the desired localized cortical damage in a CCI experiment. A similar consideration holds for the assessed ranges of the two modeling parameters, namely the thickness of the DAM shell elements and the friction between the DAM and the probe.

The choice of a neo-Hookean material model was dictated by the similarity of the loading scenario between the material calibration tests (MacManus et al., 2017) and the CCI experiments considered here. Nevertheless, there exist other more refined hyperelastic material model definitions (e.g., Mooney–Rivlin, Ogden) which might better capture the asymmetry in the tension-compression response of the tissues (de Rooij and Kuhl, 2016; Zhao et al., 2018; Budday et al., 2019). Provided that these constitutive laws are calibrated with experimental loading conditions that are similar to a CCI, future studies should include the material model as an input variable for the sensitivity analysis and assess the effect of the uncertainty of the hyper-viscoelastic parameters on the injury metrics. Another possible extension of this study should regard the assessment of the effect of the location of the point of impact on the DAM surface on the brain’s mechanical response. However, in order to assess the influence of the impact location, the limitation of the current FE model in considering a scaled average brain must be first overcome. These analyses could be repeated using subject-specific FE brain models with geometry obtained from high-resolution images, which were not available for this study. This could bring more insight, since these models could then capture the accurate morphology of the superficial gyral pattern, as it can alter the strain and the strain rate pattern (Ho and Kleiven, 2009). Finally, it is worth mentioning that the importance ranking of the input parameters refers to the brain’s response localized around the area that is directly affected by the CCI. The influence of the parameters on the spatial distribution of the whole brain strains and strain rates might differ; however, this was outside of the scope of our study.

### CC Tissue-Level Injury Metrics

The use of FE head models to investigate the mechanical tolerances associated with specific traumatic brain injuries is regarded as a promising area of brain biomechanics research with potential applications in the engineering and optimization of protective devices such as helmets, as well as in forensics. With the current investigation, we assessed the injury prediction performance of four strain and strain rate-based injury metrics for cerebral contusion using *in silico* porcine brain models.

Given the very localized injury pattern resulting from CCI, the use of strain and strain rate injury criteria has been preferred over other metrics assessed in the literature, such as those based on stress, intracranial pressure, or strain energy. Interestingly, the uncertainty analysis highlights that MPS exhibits, indeed, the least variability in comparison with the other injury metrics (Figure 4). Mao and Yang (2011) developed a FE model of the rat brain undergoing a CCI and confirmed the predictive capability of maximum principal strain and maximum shear strain as injury thresholds, finding no correlation between CC and the intracranial pressure. This can be ascribed to the fact that CCI affects only a limited area in proximity of the brain surface, and it is different than the coup-contrecoup contusions that result from impacts where the global head kinematics are involved.

Among the injury metrics considered, only the strain-based indicators were tested as being statistically significantly effective in distinguishing between injury and no injury cases. The LOOCV model evaluation confirmed the superior robustness and performance of MPS and MSS compared to MPSR and MPSXSR in terms of predicting CC. This suggests that the mechanical deformation pattern of the brain tissue—rather than its rate of deformation—shows potential in predicting focal TBI. Despite the small size of the CC injury dataset, the 50% injury risk thresholds are in line with the values reported in the literature, even though from different animal models. Our estimates of the MPS and MSS-based thresholds (0.16 and 0.22, respectively) are close to the 0.265 MPS and 0.281 MSS reported by Mao and Yang (2011) who performed CCI on rat brains. An engineering strain of 0.19 was suggested by Viano and Lövsund (1999) for 50% risk of contusion, after analyzing the results of CCI tests on ferrets. They also determined the threshold for the product of strain and strain rate as equal to 30.7 s^−1^, which is in the neighborhood of the value estimated here (MPSXSR = 45 s^−1^). Nevertheless, the interpretation of the comparison across species needs to be done with caution, given the considerable anatomical and structural brain differences. To the best of the authors’ knowledge, no other studies reported CC injury thresholds obtained by analyzing data from CCI experiments *in vivo* on pig brains.

An evident limitation of this study is the small size of the experimental dataset, which yields broad confidence intervals for the estimated CC injury thresholds, thus discouraging the use of these values as ultimate CC criteria. It is nevertheless unrealistic to presume the existence of a single injury metric capable of predicting with absolute certainty the risk of CC resulting from any loading scenario. Indeed, the use of injury metrics based on FE-derived mechanical quantities hypothesizes a direct relationship between the outcome of a TBI and the biomechanical response of brain tissue. Even in a well-controlled testing scenario as a CCI, this inevitably excludes all the complex pathophysiological mechanisms typical of secondary injury responses, which can arise during the 48 h considered by De Kegel et al. (2021) for the postimpact contusion evaluation. These biological processes are not pathognomonic to CC and depend on the genetic variability between animals. It is also unknown how they influence the targeted injury outcome, which complicates the establishment of an injury threshold and therefore represents a shortcoming. Nevertheless, a scan taken immediately following the impact could be interpreted as falsely negative. Therefore, the only objective way to determine the actual cerebral contusion damage was a binary assessment of disruption vs. no disruption of the thin cortical layers 48 h post-CCI.

Machine learning-based algorithms (e.g., deep neural networks, support vector machine, and random forest classifiers) have been regarded as promising tools to predict TBI in a more accurate and effective way (Hernandez et al., 2015; Cai et al., 2018; Wu et al., 2018; Wu et al., 2020). The advantage of these techniques is that the injury classification is performed utilizing multiple features such as elementwise brain strain. On the contrary, the FE-based scalar metrics considered here *via* the predictor-specific LOOCV suffer from information loss, since they basically reduce the complex mechanical response of the brain to a single value. Provided that a larger injury vs. no injury dataset is available, future studies should consider the evaluation of machine learning feature-based CC predictors, which can overcome the limitation of the use of single value injury metrics.

## Conclusion

We investigated the effect of common experimental and computational variables on the mechanical response of the porcine brain undergoing CCI. We successfully developed a machine learning surrogate of the finite element porcine brain model to perform the uncertainty and sensitivity analyses in a computationally efficient way. We observed that the depth and velocity of indentation should be chosen carefully when designing CCI experiments as they produce the largest influence on the brain tissue’s deformation and rate of deformation. Four strain and strain rate-based criteria have been evaluated as injury metrics to predict cerebral contusion. The maximum principal strain and maximum shear strain were found to be good candidates as tissue-level metrics specific for cerebral contusion. We demonstrated that the proposed blended *in vivo-in silico* methodology shows potential in predicting CC, although the reliability depends on the size of the animal experimental injury vs. no injury dataset. Accurate CC injury classification methods and tolerances will find practical application in the development of safer protective headgear and in forensics.

## Data Availability

The original contributions presented in the study are included in the article/Supplementary Material; further inquiries can be directed to the corresponding author.
