# Supplementary material for: A Machine Learning Approach to Investigate the Uncertainty of Tissue-Level Injury Metrics for Cerebral Contusion
Source: Front Bioeng Biotechnol. 2021 Oct 8;9:714128. doi: 10.3389/fbioe.2021.714128 (PMC8531645; doi:10.3389/fbioe.2021.714128)

Supplementary Material

# Supplementary Tables

**Supplementary Table 1**. Sensitivity indices (±95% confidence interval) *S1* and $\delta$ of MPS, MPSR, MPSXSR, MSS as a function of the seven FE model input parameters. The sensitivity analysis was carried out with 10 000 samples.

| **Input parameter** |  | **δ** | **S1** |
| --- | --- | --- | --- |
| Diameter probe | *MPS* | 0.063±0.003 | 0.036±0.007 |
|  | *MPSR* | 0.167±0.004 | 0.148±0.010 |
|  | *MPSXSR* | 0.135±0.008 | 0.120±0.011 |
|  | *MSS* | 0.060±0.003 | 0.031±0.007 |
| Velocity of indentation | *MPS* | 0.176±0.006 | 0.208±0.010 |
|  | *MPSR* | 0.372±0.008 | 0.546±0.015 |
|  | *MPSXSR* | 0.341±0.007 | 0.453±0.011 |
|  | *MSS* | 0.146±0.009 | 0.147±0.009 |
| Depth of indentation | *MPS* | 0.414±0.008 | 0.683±0.008 |
|  | *MPSR* | 0.162±0.008 | 0.159±0.012 |
|  | *MPSXSR* | 0.202±0.006 | 0.209±0.010 |
|  | *MSS* | 0.477±0.007 | 0.772±0.004 |
| Inclination of the probe | *MPS* | 0.021±0.003 | 0.002±0.002 |
|  | *MPSR* | 0.033±0.005 | 0.001±0.003 |
|  | *MPSXSR* | 0.046±0.004 | 0.001±0.003 |
|  | *MSS* | 0.022±0.003 | 0.002±0.002 |
| DAM-probe friction | *MPS* | 0.023±0.006 | 0.003±0.002 |
|  | *MPSR* | 0.030±0.003 | 0.002±0.002 |
|  | *MPSXSR* | 0.045±0.003 | 0.002±0.002 |
|  | *MSS* | 0.020±0.003 | 0.003±0.002 |
| Thickness DAM | *MPS* | 0.025±0.004 | 0.002±0.003 |
|  | *MPSR* | 0.028±0.004 | 0.002±0.002 |
|  | *MPSXSR* | 0.042±0.005 | 0.002±0.001 |
|  | *MSS* | 0.026±0.007 | 0.003±0.004 |
| Shape of the tip of the probe | *MPS* | 0.040±0.008 | 0.020±0.007 |
|  | *MPSR* | 0.059±0.005 | 0.030±0.005 |
|  | *MPSXSR* | 0.061±0.003 | 0.017±0.007 |
|  | *MSS* | 0.025±0.004 | 0.007±0.004 |

# Supplementary Figures

## ROC-training

**Supplementary Figure 1.1**. Receiver operating characteristic (ROC) curves for the training dataset considering the maximum principal strain (MPS) predictor. The colored curves represent the ROC of the training dataset of each iteration in the leave-one-out cross-validation.
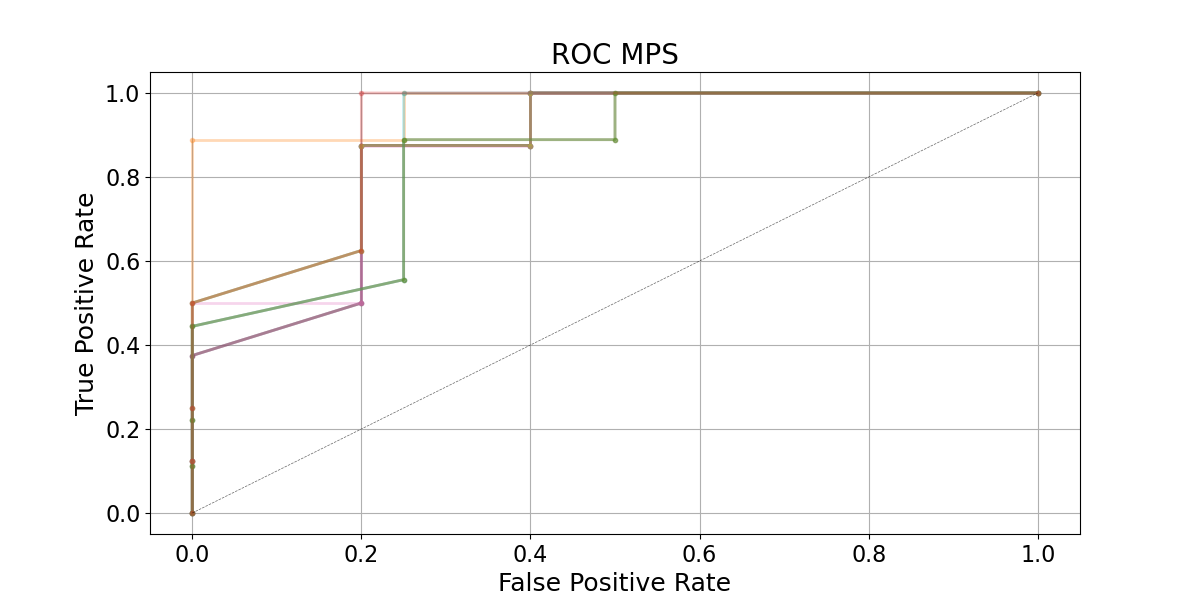


**Supplementary Figure 1.2**. Receiver operating characteristic (ROC) curves for the training dataset considering the maximum principal strain rate (MPSR) predictor. The colored curves represent the ROC of the training dataset of each iteration in the leave-one-out cross-validation.
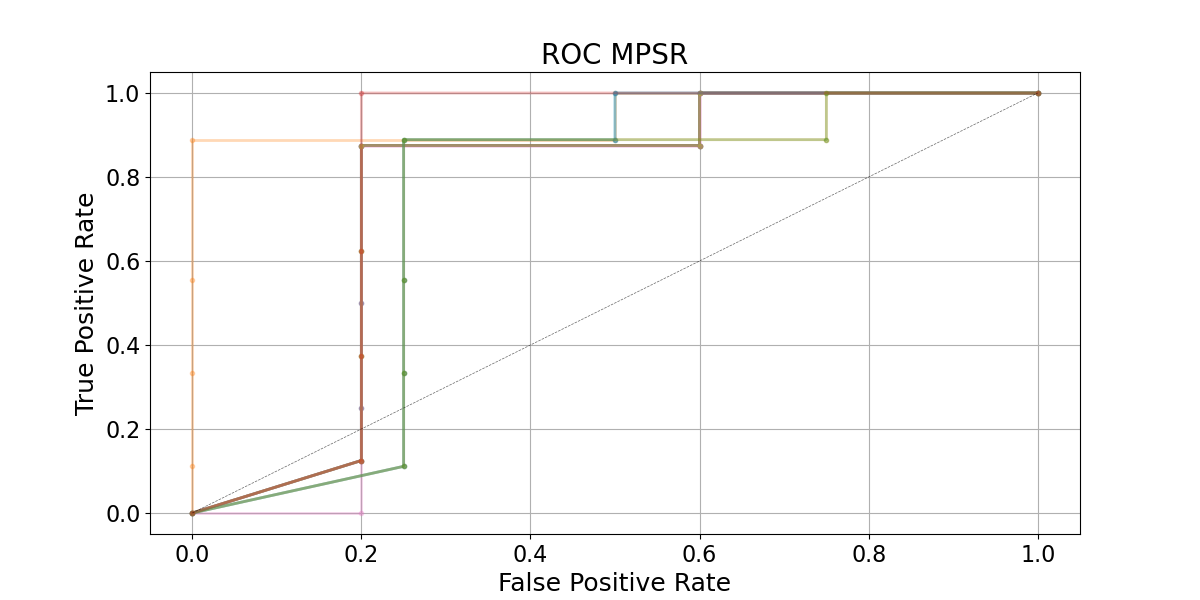


**Supplementary Figure 1.3**. Receiver operating characteristic (ROC) curves for the training dataset considering the maximum principal strain * strain rate (MPSXSR) predictor. The colored curves represent the ROC of the training dataset of each iteration in the leave-one-out cross-validation.
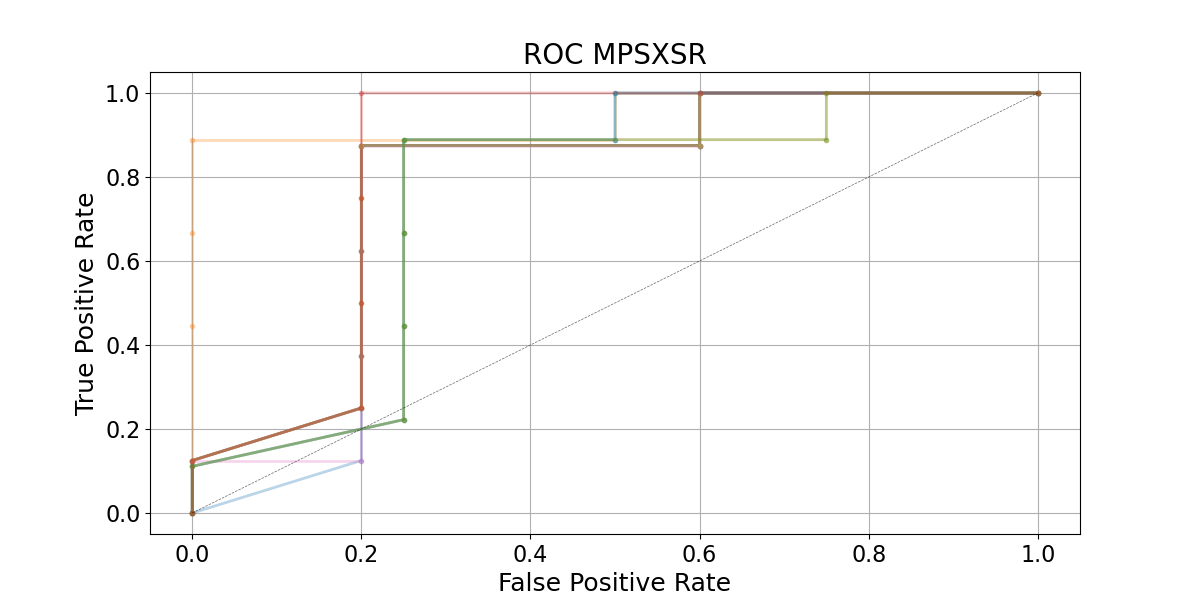


**Supplementary Figure 1.4**. Receiver operating characteristic (ROC) curves for the training dataset considering the maximum principal shear strain (MSS) predictor. The colored curves represent the ROC of the training dataset of each iteration in the leave-one-out cross-validation


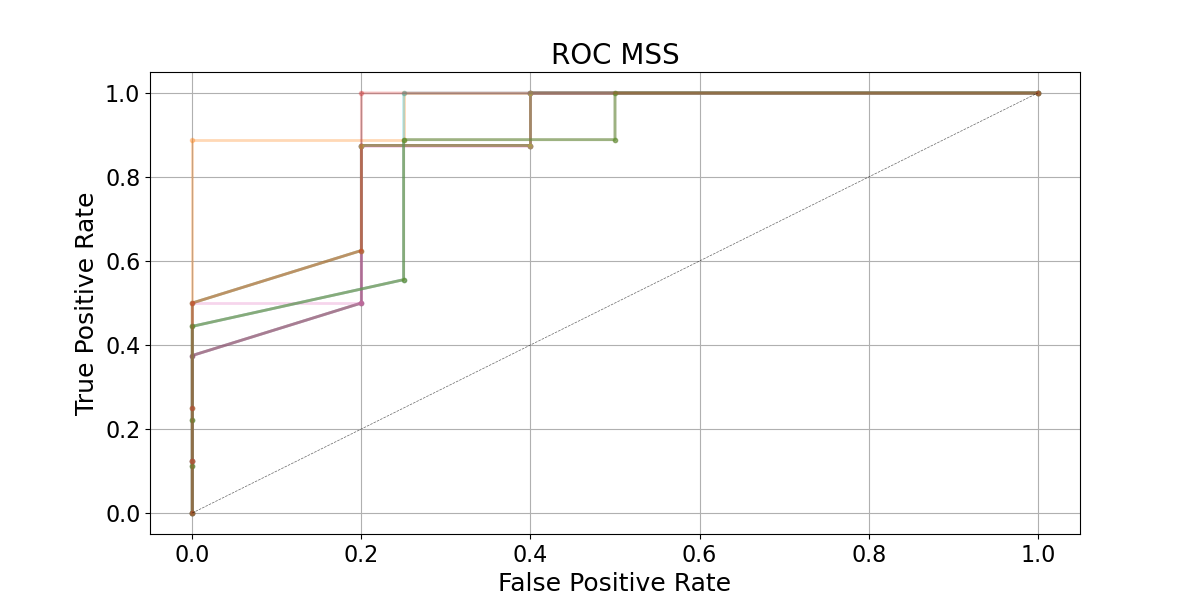


## ROC-testing

**Supplementary Figure 2.1**. Receiver operating characteristic (ROC) curve for the testing dataset considering the maximum principal strain (MPS) predictor.
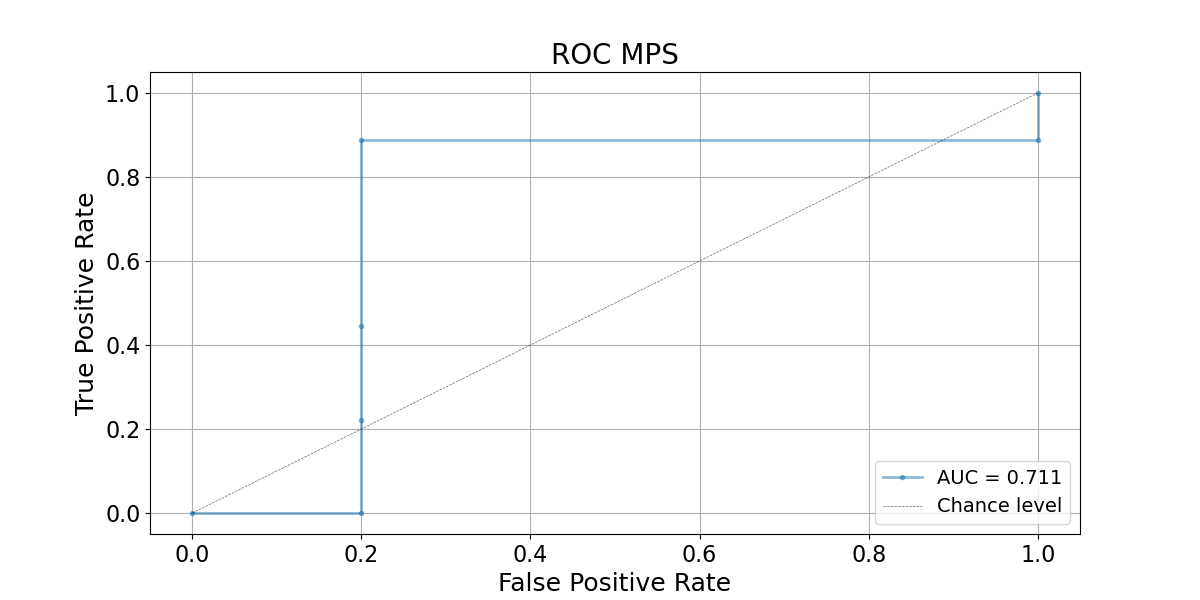


**Supplementary Figure 2.2**. Receiver operating characteristic (ROC) curve for the testing dataset considering the maximum principal strain rate (MPSR) predictor.
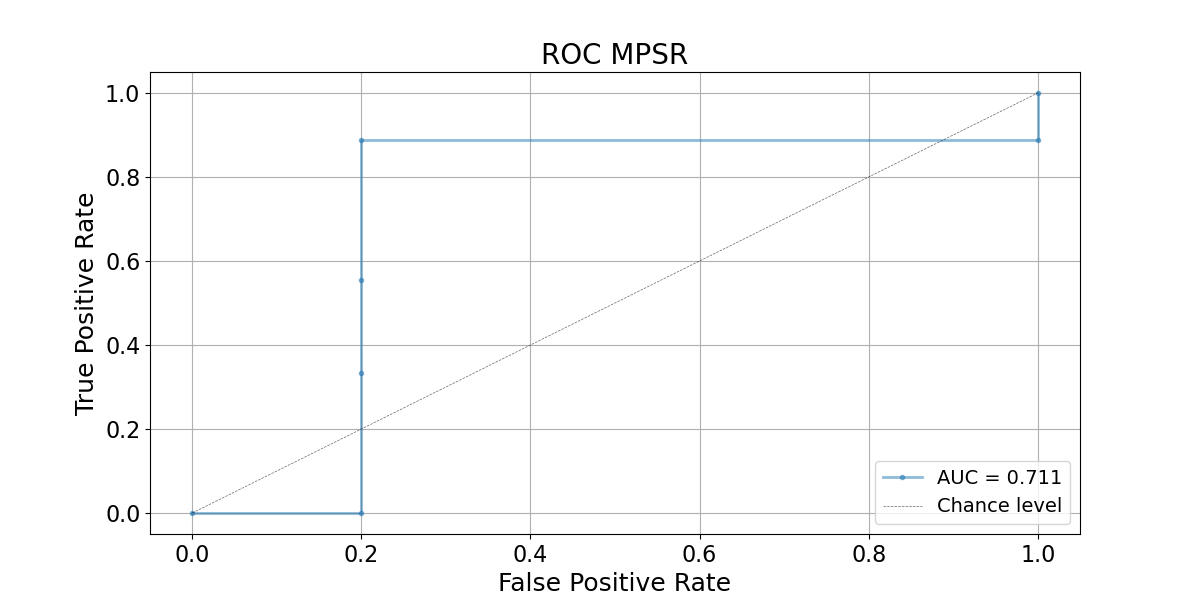


**Supplementary Figure 2.3**. Receiver operating characteristic (ROC) curve for the testing dataset considering the maximum principal strain * strain rate (MPSXSR) predictor.
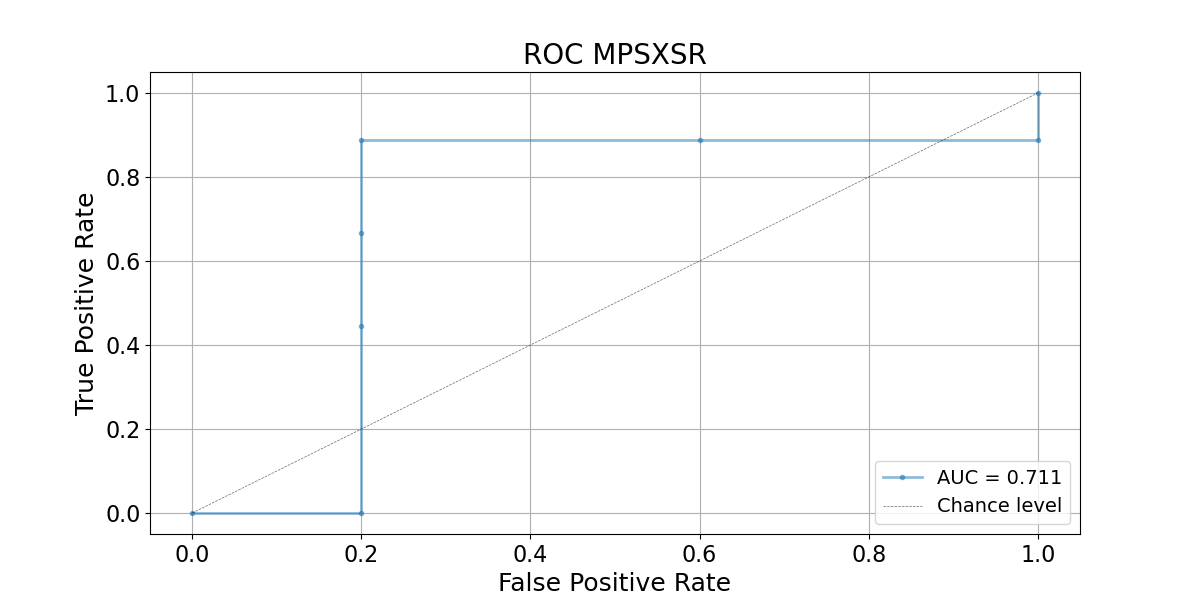


**Supplementary Figure 2.4**. Receiver operating characteristic (ROC) curve for the testing dataset considering the maximum shear strain (MSS) predictor.
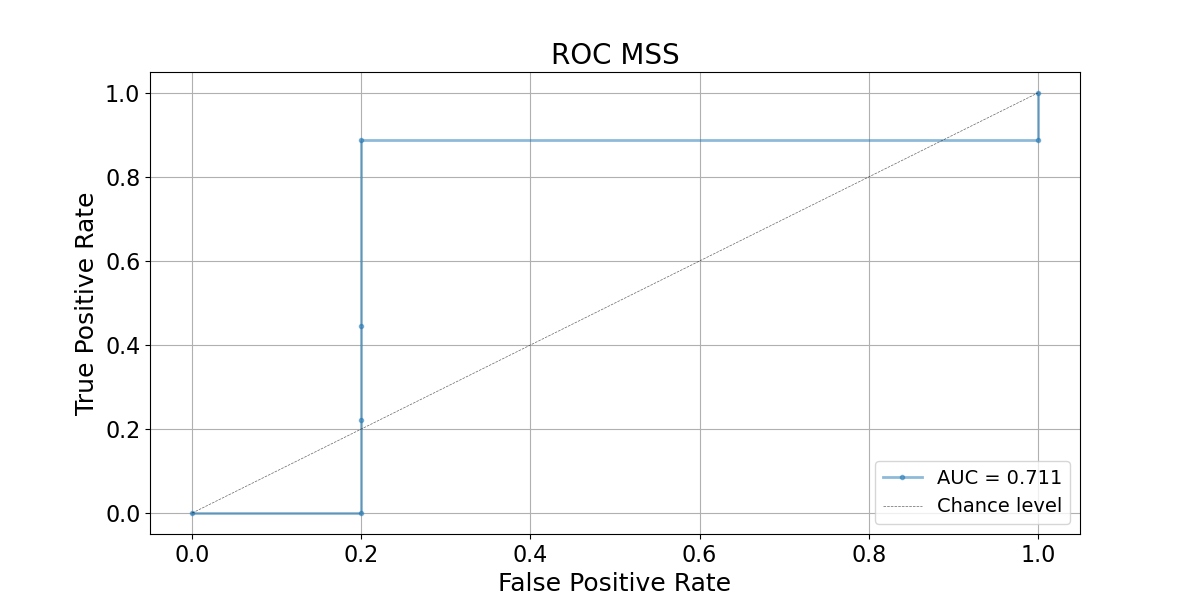

Supplement: Supplementary file 1 [file Table1.DOCX]
